# M6A-Related Long Non-Coding RNA Displays Utility in Predicting Prognosis, Portraying the Tumor Immune Microenvironment and Guiding Immunotherapy in Pancreatic Ductal Adenocarcinoma

**DOI:** 10.3390/vaccines11030499

**Published:** 2023-02-21

**Authors:** Guangyu Xu, Yutian Ji, Lufeng Wang, Hao Xu, Chaodong Shen, Haihao Ye, Xiangchou Yang

**Affiliations:** 1 Department of Hematology and Medical Oncology, The Second Affiliated Hospital and Yuying Children’s Hospital of Wenzhou Medical University, Wenzhou 325000, China; 2 Zhejiang University School of Medicine, Hangzhou 310030, China; 3 Neurology Department, East Hospital, Tongji University, Shanghai 310000, China; 4 Department of Neurosurgery, The First Affiliated Hospital of Wenzhou Medical University, Wenzhou 325000, China; 5 Department of Cardiology, Wenzhou Traditional Chinese Medicine Hospital, Wenzhou 325000, China

**Keywords:** N6-methyladenosine, lncRNA, pancreatic ductal adenocarcinoma, tumor immunology, tumor immune microenvironment, immunotherapy, immune checkpoint blockade

## Abstract

N6-methyladenosine (m6A) lncRNA plays a pivotal role in cancer. However, little is known about its role in pancreatic ductal adenocarcinoma (PDAC) and its tumor immune microenvironment (TIME). Based on The Cancer Genome Atlas (TCGA) cohort, m6A-related lncRNAs (m6A-lncRNA) with prognostic value were filtered using Pearson analysis and univariate Cox regression analysis. Distinct m6A-lncRNA subtypes were divided using unsupervised consensus clustering. Least absolute shrinkage and selection operator (LASSO) Cox regression was applied to establish an m6A-lncRNA-based risk score signature. The CIBERSORT and ESTIMATE algorithms were employed to analyze the TIME. The expression pattern of TRAF3IP2-AS1 was examined using qRT-PCR. The influence of TRAF3IP2-AS1 knockdown on cell proliferation was estimated by performing CCK8, EdU and colony-formation assays. Flow cytometry was applied to measure the effect of TRAF3IP2-AS1 knockdown on cell cycle and apoptosis. The in vivo anti-tumor effect of TRAF3IP2-AS1 was validated in a tumor-bearing mouse model. Two m6A-lncRNA subtypes with different TIME features were clarified. A risk score signature was constructed as a prognostic predictor based on m6A-lncRNAs. The risk score also correlated with TIME characterization, which facilitated immunotherapy. Finally, the m6A-lncRNA TRAF3IP2-AS1 was proved to be a tumor suppressor in PDAC. We comprehensively demonstrated m6A-lncRNAs to be useful tools for prognosis prediction, TIME depiction and immunotherapeutic guidance in PDAC.

## 1. Introduction

Pancreatic ductal adenocarcinoma (PDAC) is a fatal disease characterized by high malignancy and poor prognosis. Although the incidence is relatively low, it is still a serious threat to health globally, with only a 10% five-year survival rate [1]. According to related statistics, pancreatic cancer might achieve second place in the cancer mortality rankings before 2030 [2]. Most patients with PDAC miss the best opportunity for early diagnosis and treatment because of its indolent progression. At diagnosis, only 10–15% of PDAC patients are eligible to undergo radical surgical resection, which is the current primary treatment option [3]. Furthermore, most patients with PDAC experience metastasis, which makes the disease difficult to cure [4]. Developing effective methods for eliminating PDAC has long been a hot topic of study. In addition to surgical treatment, other types of therapies have been proposed, such as neoadjuvant therapy, targeted therapy and immunotherapy, which, unfortunately, have only benefited a small number of PDAC patients [5,6]. Therefore, there is an urgent need to develop new biomarkers and novel targets for anti-PDAC therapy while deepening our understanding of PDAC progression.

PDAC behaves like a horrible devil hiding behind hard shields; that is to say, PDAC tumors are often surrounded by a unique tumor immune microenvironment (TIME). The PDAC TIME has two prominent characteristics: (1) a high degree of desmoplasia and (2) the massive presence of immunosuppressive cells [7,8]. Stromal components strongly contribute to the development of PDAC and endow the tumors with significant resistance against anti-PDAC therapies [9,10]. Extracellular matrix proteins in the TIME can block the delivery of anti-cancer modalities [11,12,13]. In addition to the physical barriers, TIME components can also neutralize the effects of chemotherapeutic agents through biological pathways such as the SDF-1/SATB-1 axis [14]. Moreover, immunosuppressive effects (such as the exhaustion of cytotoxic T-cells activity) caused by interactions among tumor-infiltrating immune cells have been observed within lesions, which might account for the poor response to immunotherapies [7,8,15,16]. TIME components also contribute to PDAC metastasis [17]. Collectively, these effects from the TIME might lead to treatment failure in clinical practice. Recently, there have been attempts to treat PDAC by targeting the TIME [18,19]. Research taking TIME components as new stratification markers has also been carried out [20]. Precursory clinical trials have been conducted, but the outcomes were not satisfactory [9,21,22,23,24], suggesting that utilizing TIME as a novel target for PDAC diagnosis and treatment still has difficulties to overcome. Currently, the mysterious veil around the PDAC TIME has not been completely lifted. Breaching this hard shell is critical for conquering PDAC. Therefore, efforts to find new research perspectives and targets for the PDAC TIME are urgently needed.

Long non-coding RNAs (lncRNAs) have been regarded as “background noises of genomic transcription” due to the present lack of a comprehensive understanding. As a matter of fact, increasing research has indicated that lncRNAs could exert their biological influence on cancers at multiple levels. Several lines of evidence have revealed that lncRNAs play a pivotal role in cancer immunity development [25,26] and TIME formation [27,28]. Interestingly, recent evidence revealed that lncRNAs interact with the N6-methyladenosine (m6A) modification system, and the crosstalk generated between m6A modifications and lncRNAs contributes to carcinogenesis [29].

M6A is a pervasive form of epigenetic modification commonly seen in various kinds of RNAs [30]. This type of modification is regulated by complex interactions among demethylases, signal transducers and methyltransferases, which are also metaphorized as “erasers”, “readers” and “writers” [31]. In recent years, m6A has emerged in cancer biology as a hot topic. Related reports have highlighted that m6A is deeply involved in a variety of cancer types, including PDAC [32]. Current research has revealed that most of the m6A regulator proteins play oncogenic roles in PDAC [32,33,34,35,36]. Moreover, a few of them possess anti-tumor functions [37,38]. These research conclusions indicate the existence of a complex m6A regulation network in PDAC. However, as another important player, the relevance of the lncRNA and m6A regulation system for PDAC has been poorly investigated. The biological roles of these m6A-related lncRNAs in PDAC, especially their effects on the PDAC TIME, have not yet been fully investigated.

Therefore, in this study, by utilizing the transcriptomic data of 177 PDAC patients from TCGA, we comprehensively analyzed the patterns of m6A-lncRNA. Initially, according to the expression patterns of m6A-lncRNA, two distinct subtypes of PDAC patients were identified using unsupervised consensus clustering. Corresponding features for the TIME and biological processes were clarified. Then, we constructed a dependable m6A-lncRNA-based risk score signature for clinical evaluation and prognostic prediction. Furthermore, the TIME characteristics and biological signaling pathway activities were depicted for different risk scores. The risk score signature also demonstrated its utility in guiding immunotherapy for PDAC patients. In addition, the clinical significance and anti-tumor functions of TRAF3IP2-AS1, an m6A-lncRNA in our model, were validated both in vitro and in vivo. The findings of our research provide clinicians and researchers with new insights for PDAC treatment.

## 2. Materials and Methods

### 2.1. Sources of Information and Initial Data Handling

The transcriptomic sequencing and clinical data for PDAC patients were obtained from The Cancer Genome Atlas (TCGA) database and Genotype-Tissue Expression database (171 normal and 177 tumoral). Transcriptomic data for 23 m6A regulators were collected from the TCGA database. Data concerning copy number variations (CNVs) in PDAC were obtained from the UCSC Xena database.

### 2.2. Analyses of Genome Mutation Data

The distribution statuses of the CNVs of the 23 m6A regulators on human chromosomes were portrayed with the running R package Rcircos.

### 2.3. Analysis of Functional and Pathway Enrichment

Gene Ontology (GO) and the Kyoto Encyclopedia of Genes and Genomes (KEGG) were adopted to comprehensively reveal the genes’ biological functions across many aspects. We used the clusterProfiler R package to perform GO and KEGG analyses with a cutoff *p* < 0.05 and q < 0.05. Quantification of pathway activity was realized by applying the HALLMARK gene set. To reveal the differences in biological pathways between distinct clusters, we used gene set variation analysis (GSVA) by launching GSVA R packages.

### 2.4. Identification of Outcome-Related m6A-lncRNAs

The m6A-lncRNAs were filtered by performing a Pearson correlation analysis. The criteria for m6A-lncRNA identification were a correlation coefficient |R| > 0.4 and *p* < 0.001. Outcome-related lncRNAs were selected with the help of univariate Cox regression analysis. Forest plots were drawn to exhibit the outcomes of univariate Cox regression analysis. Difference analysis of expression levels was then carried out. The overall results of the difference analysis were presented in heatmap and boxplot forms. The data were processed using the R pheatmap and ggpubr packages.

### 2.5. Consensus Clustering of Outcome-Relevant lncRNAs

In accordance with the best cutoff value (k = 2), PDAC cases were clustered into distinct subgroups by running the NMF R package. With the ConsensusClusterPlus R package, unsupervised clustering analysis was implemented. Kaplan–Meier (K-M) curves were drawn to reveal the difference in survival between these two clusters.

### 2.6. Depiction and Comparison of the TIME between Clusters

The CIBERSORTx algorithm was applied to reveal the infiltration status of various immunocytes in tumoral lesions. A total of 22 infiltrated immunocytes in PDAC samples were quantified using CIBERSORTx. Expression data (ESTIMATE) analysis was performed to measure the differences in the TIME by running the estimate R package. The differences in the TIME between distinct clusters were calculated using the Wilcoxon rank sum test.

### 2.7. Establishment and Validation of an m6A-lncRNA-Based Prognostic Risk Score Signature

The division of training and testing sets was completed with the R project caret package with a ratio of 3:2 [39]. The LASSO Cox regression was implemented with the help of the glmnet R package. The formula for the final risk score calculation was as follows:

$$\mathrm{Risk}\;\mathrm{score}=\sum_{i=1}^{n}Coefi\times xi$$

where *Coefi* is the coefficient, and *xi* is the TPM value of every m6A-lncRNA.

The PDAC patients in different sets (training set and validation set) were classified into two groups according to the median value of the risk score. The survival situation of each group was revealed using Kaplan–Meier analysis. By running the R package timeROC, receiver operating characteristic (ROC) curves were drawn and the area under curve (AUC) values were measured to calculate the efficacy for predicting one-, two- and three-year outcomes.

### 2.8. Cell Lines and Cell Culture

Five PDAC cell lines (PANC-1, MIA-Paca 2, SW1990, BxPC-3 and CFPAC) were kept at our laboratory. The cells were maintained in an incubator (Heracell™ Vios 160i CR CO_2_ Incubator, Thermo Fisher Scientific, Waltham, MA, USA) with a standard pathogen-free environment (humidified, 37 °C, 5% CO_2_). PANC-1 cells, MIA-Paca 2 cells and SW1990 cells were cultured in Dulbecco’s minimal essential medium (DMEM). BxPC-3 cells and CFPAC cells were cultured in Rosewell Park Memorial Institute-1640 medium (RPMI-1640). All of the cell culture media contained 10% fetal calf serum (Gibco, Thermo Fisher Scientific, Waltham, MA, USA). Furthermore, antibiotics (penicillin and streptomycin) were also added into the media under the proper conditions (100 U/mL for penicillin; 100 µg/mL for streptomycin). When the degree of cell fusion reached approximately 90%, the cells were dissociated and harvested with the help of trypsin. Then, the cells were passaged properly using standard techniques.

### 2.9. Isolation of Total RNAs and Implementation of Quantitative Real-Time PCR

Total RNAs were collected and purified by utilizing an RNA-Quick Purification Kit (Shanghai YISHAN Biotechnology, RN001, Shanghai, China). The synthesis of cDNA templates was completed via reverse transcription with proper reagents from a HiScript^®^ III RT SuperMix Kit for qPCR (Vazyme, RC323-01, Nanjing, China). A standard qRT–PCR procedure was operated on a PCR instrument (QuantStudio 7 Flex, Applied Biosystems, Foster City, CA, USA). The reagents for qRT-PCR were obtained from a TB Green^®^ Premix Ex Taq™ II (Tli RnaseH Plus) Kit (Takara, RR820A, Japan). The specific primer sequences for qRT-PCR are listed in the additional file.

### 2.10. The TRAF3IP2-AS1 Knockdown Model

The knockdown was realized by transfecting the PDAC cells with small interfering RNA (siRNA) and small hairpin RNA (shRNA) (Tsingke Biotechnology Co., Ltd. Beijing, China) against TRAF3IP2-AS1. The siRNA transfection was accomplished with the assistance of Lipofectamine™ 3000 (Invitrogen, Thermo Fisher, Waltham, MA, USA) following the standard protocols. For the in vivo knockdown model, the PANC-1 cells were transfected with shRNA. Lentiviruses were used as vectors for importing the shRNA into cells. The stable cell line was filtered with puromycin (Invitrogen, Thermo Fisher, Waltham, MA, USA) at a concentration of 3 μg/mL. Finally, the knockdown efficacy was validated by running qRT-PCR. The specific sequences of siRNA and shRNA are listed in Supplementary Tables S1–S3.

### 2.11. The Estimation of Cellular Proliferative Ability

In summary, the proliferative ability of cells was estimated using the Cell Counting Kit-8 assay (CCK-8 assay), 5-ethynyl-2′-deoxyuridine (EdU) assay and colony-formation assay. For the CCK-8 assay, the CCK-8 reagents were purchased from Dojindo Laboratories, Kumamoto, Japan. At the time point of 48 h after siRNA transfection, cells in the logarithmic growth phase were dissociated from the cell culture dishes, resuspended and planted into 96-well plates at a density of 4000 cells per well. The CCK-8 reagents were added to the wells to measure the proliferation status of the cells. The absorbance at 450 nm was measured daily using a multifunctional microplate reader (TECAN SPARK, Männedorf, Switzerland) over four consecutive days. For the EdU assay, cells were harvested, fixed and stained with reagents from a BeyoClick™ EdU Cell Proliferation Kit with Alexa Fluor 594 (Beyotime, Shanghai, China). The fluorescent signals were observed through a ZEISS LSM 900 confocal microscope (LSM 900, ZEISS, Oberkochen, Germany). Fluorescent images were taken and proliferative statuses were analyzed. For the colony-formation assay, we performed the assay by seeding transfected cells into six-well plates with 600 cells per well over 10–14 consecutive days. A clone was defined as at least 50 assembled cells. The cells were stained with crystal violet (cat. no. C0121, Beyotime, Shanghai, China), and the number of clones was counted and recorded.

### 2.12. Flow Cytometric Analysis of Cell Apoptosis

The apoptotic status of the cells was detected using flow cytometry. At the timepoint of 48 h after siRNA transfection, cells were dissociated and collected. Then, the cells were double-stained with fluorescent reagents in an AnnexinV-FITC/Propidium Iodide Apoptosis Detection Kit (cat. no. C1062, Beyotime, Shanghai, China). After staining, the fluorescent signals of each cell were detected and recorded with a BD Beckman cytometer (BD Biosciences, Franklin Lake, NJ, USA). When running flow cytometric analyses, the fluorescent signal of propidium iodide was detected via the PE channel, and the fluorescent signal of Annexin V-FITC was detected via the FITC channel. The data were then exported and, finally, analyzed using FlowJo software (version 7.6.1). The results of the flow cytometric analysis were gated quadrants, and the overall apoptosis rates were calculated by summing the early-phase apoptosis percentage (Q3 quadrant) and late-phase apoptosis percentage (Q2 quadrant).

### 2.13. Flow Cytometric Analysis of Cell Cycle

The cells were collected 48 h after transfection. To fix the cells, 70% ethanol was used. The fixed cells were stored at 4 ℃ for over 24 h and then stained using reagents from a Cell Cycle and Apoptosis Analysis Kit (cat. no. C1052, Beyotime, Shanghai, China). Cell cycle distribution was detected by operating flow cytometry. The data were analyzed and visualized using ModFit software.

### 2.14. Detection of Caspase 3 and Caspase 9 Activity

The caspase 3 and caspase 9 activities were detected using a Caspase 3 Activity Assay Kit (cat. no. C1116, Beyotime, Shanghai, China) and a Caspase 9 Activity Assay Kit (cat. no. C1158, Beyotime, Shanghai, China). Following the instructions attached to the kits. The cells were lysed and centrifuged at 18,000g for 15 min. The supernatant was collected and mixed with lysis buffer and substrates (Ac-DEVD-pNA for caspase 3, Ac-LEHD-pNA for caspase 9). The mixture was then added into a 96-well plate and incubated at 37 °C for 60 min (or overnight if necessary). After that, the 450 nm OD values were detected using a multifunctional microplate reader. Finally, the caspase 3 and caspase 9 activities were quantified according to the pNA standard curve.

### 2.15. Xenograft Model and In Vivo Validation

A batch of five-week-old BALB/c nude mice (weighing 20 g) was purchased. The mice were fed under standard SPF conditions. The PANC-1 cells (5 × 10^6^ per mouse) were injected subcutaneously into the right flank regions of the mice. After 21 days of growth, the mice were euthanized and tumor tissue samples were collected. The tumors were fixed in formalin, embedded in paraffin and stained with eosin and hematoxylin (i.e., HE staining). All animal research complied with protocols approved by the Laboratory Animal Centre, Wenzhou Medical University.

### 2.16. Immunohistochemistry (IHC)

Tumor samples from the mice were formalin-fixed, paraffin-embedded, sectioned and incubated with a primary mouse Ki-67 antibody diluted at a concentration of 1:400 (Abcam, Cambridge, UK). When the staining was completed, we observed the sections under a microscope (Leica, Weztlar, Germany). The Ki-67 results were estimated and quantified using the H-score. The calculation formula was H-score = Σpi(i + 1), where pi stands for the percentage of positive cells and i stands for staining color intensity.

### 2.17. TUNEL Staining

Tumor samples from the mice were sliced and then stained with FITC fluorescein-dUTP by utilizing a One Step TUNEL Apoptosis Assay Kit (Beyotime, Shanghai, China). When the staining was completed, the sections were observed through a confocal microscope. Images were taken and exported for analysis. The results of the TUNEL staining were quantified by TUNEL^+^ rates.

### 2.18. Statistical Analysis

All of the mathematical calculations, statistical analyses and graphical drawings were accomplished with the assistance of R software (4.1.3), IBM SPSS Statistics 25 and GraphPad Prism (8.3.0). The results are exhibited as the means ± standard deviation from at least three independent experiments (* means *p* < 0.05; ** means *p* < 0.01; ns, not statistically significant). All of the *p* values are two-tailed, and *p* < 0.05 was defined as statistically significant.

## 3. Results

### 3.1. Depiction of m6A Regulator Abnormality and Recognition of m6A-Related lncRNA in PDAC Patients

**Figure 1:**
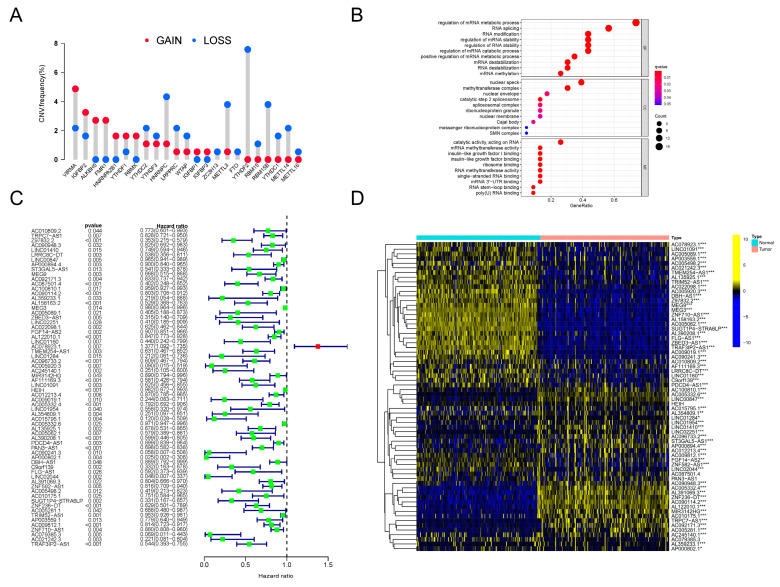
CNV analysis of m6A regulators and identification of prognosis-related m6A-lncRNA. (**A**) The frequency of CNV mutation analysis revealing the pervasive existence of genetic abnormality in all 23 m6A regulators in PDAC patients. The height of the column represents the mutation frequency. Red dots, amplification frequency; blue dots, deletion frequency. (**B**) Results of GO annotation analysis revealing the significantly correlated biological processes of 23 m6A regulators. The terms of biological processes are listed along the Y-axis, and the corresponding gene ratios are labeled on the X-axis. The bubble size stands for the number of enriched genes and the color depth stands for the degree of correlation significance. (**C**) The results of univariate Cox regression analyses were visualized in a forest plot (**D**). Heatmap presenting the differences in the expression level distributions of prognosis-related m6A-lncRNAs between normal samples (n = 171) and tumor samples (n = 177). * *p* < 0.05, ** *p* < 0.01, *** *p* < 0.001.

Firstly, we investigated the genetic variation status of m6A regulators in PDAC. An assembly of 23 m6A regulators was profiled (Table 1). We found that, in PDAC patients, copy number variations (CNVs) existed pervasively in these regulator genes. Among these regulators, ALKBH5, FMR1, HNRNPA2B1 and RBMX showed high degrees of amplification in CNV, while YTHDF2, RBM15, RBM15B, YTHDC1, METTL14 and METTL16 presented obvious deletions in CNV (Figure 1A). In addition, significant differences in the expression levels of all 23 m6A regulators between PDAC lesions and normal tissues were discovered (Supplementary Figure S1A). The expression patterns of several critical regulators were validated in clinical tissue specimens (Supplementary Figure S1B). All of this evidence indicated that the m6A regulator system was obviously abnormal and disordered in PDAC patients.

Next, to clarify further the downstream biological functions that these abnormal regulators were involved in, GO annotation analysis was orchestrated. As clearly presented, the m6A regulators were closely related to RNA metabolism, RNA splicing and RNA modification (Figure 1B). Therefore, we tried to determine the inner relationship between lncRNAs and the m6A modification system in PDAC. Following the instructions for gene ensemble IDs on the TCGA website, data for lncRNA expression in PDAC were retrieved and gathered; transcriptomic data for these m6A regulator genes were also searched and downloaded. To sum up, a total of 8724 lncRNAs were recognized. We then conducted Pearson correlation analysis to filter the lncRNAs, which showed close relationships with the dysregulated m6A regulators in PDAC. Surprisingly, by using this method, a total of 173 m6A-related lncRNAs (m6A-lncRNA) were recognized. Then, 66 lncRNAs with significant prognostic value were distinguished in this assembly using univariate Cox regression analysis. The results are presented intuitively in a forest plot (Figure 1C). By conducting difference analysis, the expression status of prognostic m6A-lncRNAs was elucidated. As can be seen in the heatmap, most of these lncRNAs were distinctly expressed in PDAC tumors compared to normal samples (Figure 1D). We finally obtained a batch of prognosis-related lncRNAs that were highly correlated with the dysregulated m6A network (defined as prognosis-related m6A-lncRNA) in PDAC, which is worth exploring in future research.

### 3.2. Depiction of PDAC TIME Features through Consensus Clustering Analysis of Prognostic m6A-lncRNA

**Figure 2:**
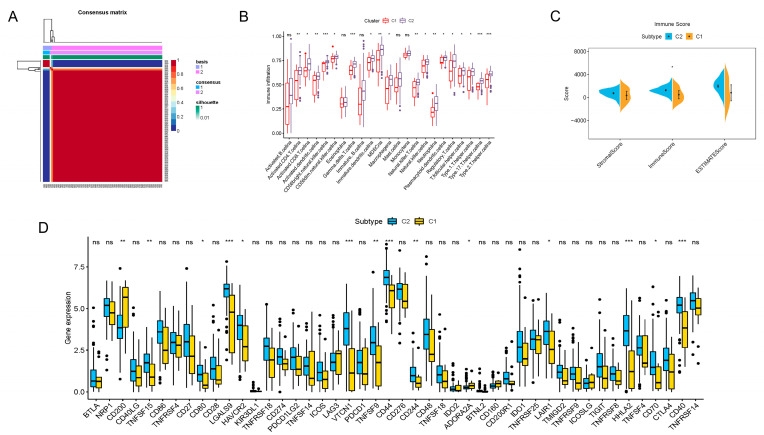
Consensus clustering of prognosis-related lncRNAs and construction of a risk score model in the PDAC cohort. (**A**) Consensus clustering matrix for k = 2. (**B**) Boxplots showing difference in immune cell infiltration between cluster 1 and cluster 2. (**C**) Results of the ESTIMATE algorithm, revealing that the main difference in the TIME between cluster 1 and cluster 2 was in the immune cell components. (**D**) Boxplots presenting results of difference analysis of multiple immune checkpoint blockade gene expression levels between cluster 1 and cluster 2. In the images, the scattered dots stands for values of gene expression and immune cell infiltration. In the boxes, the bottom borders and the top borders stand for the 25th and 75th percentiles respectively. The transversal line in the box stands for the median value. Statistical difference is expressed by asterisks, * *p* < 0.05, ** *p* < 0.01, *** *p* < 0.001, ns, not significant.

To better reveal the regular patterns underlying m6A-related lncRNA in PDAC, as well as to explore its potential in the TIME, unsupervised consensus clustering was performed on the basis of the prognostic m6A-lncRNA we identified. With the assistance of the NMF R package, we confirmed that the clustering number k = 2 was the best value for clustering stability (Supplementary Figure S2 and Figure 2A). PDAC patients were then induced into two distinct clusters (13 in cluster 1 and 164 in cluster 2) according to m6A-lncRNA expression patterns.

The immune infiltration situations in these two clusters were then analyzed and compared. By employing the CIBERSORTx algorithms, 23 major types of immunocytes commonly seen in the TIME were included in the investigation. The degree of infiltration was quantified and visualized with the algorithms. As vividly shown, a significant difference in the degree of infiltration was observed in 18 types of immunocytes (Figure 2B). Notably, the infiltration of all these critical immune cells was markedly enriched in cluster 2 in contrast to cluster 1, which indicated that PDAC patients with a cluster 1-type m6A-lncRNA pattern might possess a less activated TIME. For further clarification, a novel ESTIMATE algorithm was applied to calculate the stromal score (quantification of TIME stromal components), immune score (quantification of immune cell components) and ESTIMATE score (quantification of tumor components; namely, the tumor purity). The outcomes for distinct clusters were compared (Figure 2C). The results of the analysis revealed that the main difference in the TIMEs in the two clusters was in immune cell infiltration. Patients in cluster 2 had a higher immune score, indicating a more abundant immune cell population in the TIME, which was consistent with the findings shown in Figure 2B. In addition, differences in immune checkpoint blockade gene expression were also displayed (Figure 2D). Cluster 2 expressed higher levels of typical immune checkpoint blockade genes, such as TNFSF15, CD80, LGALS9, HAVCR2, VTCN1, TNFSF9, CD44, CD244, LAIR1, HHLA2, CD70 and CD40. Cluster 1 expressed higher levels of CD200, indicating distinct responses toward immune checkpoint blockade therapy.

In summary, we analyzed the TIME characteristics of PDAC patients through consensus clustering according to the m6A-lncRNA expression patterns. Patients in cluster 1 and cluster 2 possessed quite different TIME features, including immune cell infiltration and immune checkpoint blockade gene expression. All of these results indicate distinct responses toward immunotherapy, highlighting the excellent potential of m6A-related lncRNA in guiding PDAC immunotherapy.

### 3.3. Expression Pattern of TRAF3IP2-AS1, along with Ten Other lncRNAs, Could Be Used to Predict the Prognosis of PDAC Patients

**Figure 3:**
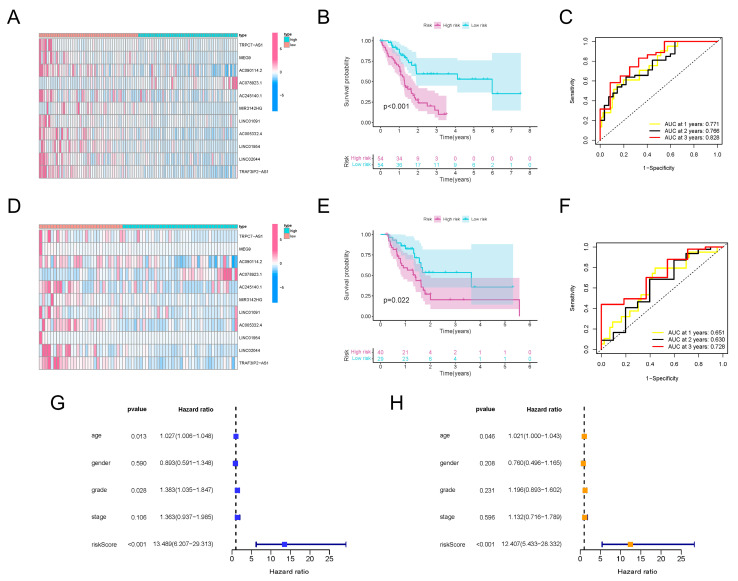
Construction and validation of m6A-lncRNA risk score signature. (**A**) Heatmap exhibiting expression values of 11 hub prognosis-relevant m6A-lncRNAs in cases from the training set. (**B**) K-M analysis clarifying different survival probabilities of PDAC patients in training set partitioned by risk score. (**C**) ROC curves validating the predictive efficacy of the m6A-lncRNA-based risk signature in the training set. (**D**) Heatmap exhibiting expression values of prognosis-related m6A-lncRNAs in cases from the testing set. (**E**) K-M analysis clarifying the survival probability of PDAC patients in the training set partitioned by risk score. (**F**) ROC curves validating the predictive efficacy of m6A-lncRNA-based risk signatures in the testing set. (**G**) The outcome of univariate independent prognostic analysis of all PDAC cases was visualized in forest plot form. The vertical dotted line represents the hazard ratio (HR) for all patients, and the length of the horizontal line shows the 95% confidential interval for each group. (**H**) The outcome of multivariate independent prognostic analysis of all PDAC cases visualized in forest plot form. The vertical dotted line represents the hazard ratio (HR) for each group, and the length of the horizontal line shows the 95% confidential interval for all PDAC patients.

Since the m6A-lncRNAs had significant prognostic value, we sought to further uncover their clinical utility. Based on the 66 prognosis-related m6A-lncRNAs, LASSO Cox regression analysis was conducted to establish a risk score model (Supplementary Figure S3A). A total of 11 m6A-lncRNAs with apparent abnormal expression patterns (TRAF3IP2-AS1, TRPC7-AS1, MEG9, AC090114.2, AC078923.1, AC245140.1, MIR3142HG, LINC01091, AC005332.4, LINC01954 and LINC02044) were selected with the optimal value for the penalty parameter λ (Supplementary Figure S3B). Risk scores for all selected PDAC samples were calculated using the formula described earlier. The median values of the risk scores were applied to classify the PDAC patients into a high-risk-score group (n = 54) and a low-risk-score group (n = 54). The expression levels of the 11 m6A-lncRNAs in the training set were investigated. Most m6A-lncRNAs in the model possessed higher abundance in the lower-risk-score group, except for AC078923.1 (Figure 3A). The partition of PDAC patients according to risk scores and their corresponding dot plots for survival time signified that the proportion who died was much smaller in the population with a low risk score (Supplementary Figure S3C). Moreover, survival analysis demonstrated that PDAC patients in the low-risk-score group had an apparent survival advantage (Figure 3B). ROC curves confirmed the dependability and excellent predictive efficacy for patients with PDAC (Figure 3C).

To verify the effectiveness of the risk score signature, a testing set consisting of 69 PDAC patients was used. The expression values for the m6A-lncRNAs in the testing set are presented in Figure 3D. The distribution of risk scores and corresponding survival times are also presented (Supplementary Figure S3D), which showed the same trend as the training set. Furthermore, the outcomes of K-M analysis also demonstrated a higher survival probability in the group with the lower risk score in the testing set (Figure 3E). The ROC curves also validated the excellent one-, two- and three-year prognostic prediction value of this model (Figure 3F). As exhibited in Figure 3G, the risk score was associated with overall survival, which indicated that it could be used as an independent predictor for PDAC prognosis. After correcting confounder factors, the risk score was still an independent predictor for PDAC patients’ overall survival (Figure 3H). Stratification survival curves for every m6A-lncRNA in the panel were drawn, revealing the fact that the abnormal expression levels of each lncRNA were also obviously related to the survival rate of PDAC patients (Supplementary Figure S4). Moreover, the model was suitable for predicting outcomes in PDAC populations under different clinicopathological conditions (Supplementary Figure S5).

All of these results demonstrate that, regardless of whether they are calculated separately or together, the expression patterns of TRAF3IP2-AS1, along with ten other lncRNAs, could be made into an effective model for prognosis prediction.

### 3.4. Signaling Pathway Enrichment Analysis and Correlation between Risk Score and TIME Characteristics

**Figure 4:**
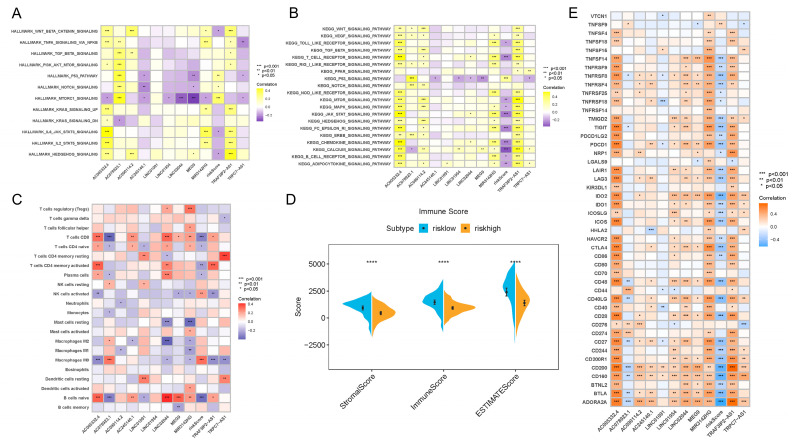
Results of enrichment analysis, TIME feature description and immunotherapy guidance using risk score signature. (**A**) Heatmap showing the representative pathway terms from Hallmark enriched in each m6A lncRNA and the risk score. (**B**) Heatmap showing the representative pathway terms from KEGG enriched in each m6A-lncRNA and the risk score. (**C**) Heatmap showing the significant positive and negative correlations between risk score and multiple infiltrative immune cells in PDAC lesions. (**D**) Results of the ESTIMATE algorithm demonstrating that patients with different risk scores were significantly different in terms of stromal score, immune score and ESTIMATE score. (**E**) Heatmap presenting the negative correlation between risk score signature and immune checkpoint gene expression in PDAC. **** *p* < 0.0001.

In order to further clarify the biological meaning of the different risk score groups in PDAC development, as well as to maximize the potential of our risk score model, we used GSVA to investigate the enriched signaling pathways. As clearly presented in Figure 4A,B, the activities of mTORC1, p53 and NOTCH signaling pathways were upregulated in PDAC patients with higher risk scores. In patients with lower risk scores, pathways such as Wnt-β-catenin, IL6-JAK-STAT3, HEDGEHOG and T cell receptor signaling were more activated. When analyzed separately, the results show that AC005332.4 and TRAF3IP2-AS1 were highly correlated with nearly all signaling pathways, which means these two lncRNAs may play critical roles in PDAC carcinogenesis and progression.

Interestingly, we also found that the risk score was associated with TIME features. It turned out that the risk score was positively related to M0 macrophage recruitment and NK cell activation, while it was negatively related to CD8^+^ T cell, naive B cell, plasma cell and CD4^+^ T cell infiltration (Figure 4C). In addition, the results of the ESTIMATE algorithm showed that the low-risk-score group and high-risk-score group were remarkably different in terms of stromal components, immune cell components and tumor cell components (Figure 4D). All of these results suggest a different composition and infiltration status for the TIME in PDAC patients with distinct risk scores.

### 3.5. The m6A-lncRNA-Based Risk Scoring Signature Was Also Effective in Guiding Immunotherapy in PDAC Patients

Next, we attempted to reveal the correlation between the risk score and immunotherapeutic responses. A series of typical genes related to immune checkpoint blockade therapy (such as PDCD-1, CTLA-4, IDO-1 and IDO-2) were found to be negatively correlated with risk score (Figure 4E). The results confirmed the inner links between risk score and immunotherapeutic responses in PDAC. Higher risk scores might mean worse responses toward immunotherapies, which might also induce worse prognosis.

### 3.6. Knockdown of TRAF3IP2-AS1 Facilitated Growth of PDAC In Vitro

**Figure 5:**
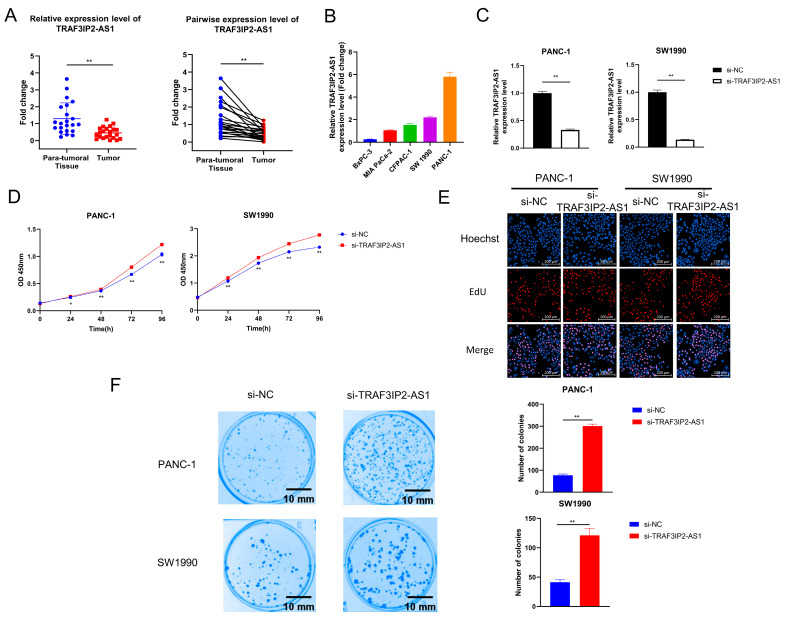
The effect of TRAF3IP2-AS1 knockdown on PDAC cell proliferation and colony formation. (**A**) The relative expression level of TRAF3IP2-AS1 in five PDAC cell lines was examined via qRT-PCR. (**B**) The results of qRT-PCR validated the efficacy of TRAF3IP2-AS1 knockdown in PANC-1 and SW1990 cells. (**C**,**D**) The influence of TRAF3IP2-AS1 knockdown on the proliferation of PANC-1 cells and SW1990 cells was estimated using CCK-8 assays (**C**) and EdU assays (**E**). (**F**) Knockdown of TRAF3IP2-AS1 improved the colony-formation ability of PDAC cells. * *p* < 0.05, ** *p* < 0.01.

During the above analyses, we noticed that TRAF3IP2-AS1 appeared to be a critical m6A-lncRNA with an obvious disordered expression pattern in PDAC. We validated its expression pattern in clinical tissue samples and found that it was downexpressed in PDAC lesions (Figure 5A). Data from TCGA also revealed that the higher expression level of TRAF3IP2-AS1 related to smaller tumor size (Supplementary Table S4).

However, the biological roles of TRAF3IP2-AS1 in PDAC remain uninvestigated. The expression levels of TRAF3IP2-AS1 in five human PDAC cell lines (BxPC-3, MIA Paca-2, CFPAC-1, SW1990 and PANC-1) were examined via qRT-PCR. The expression levels of TRAF3IP2-AS1 varied in distinct cell lines, and the BxPC-3 cells showed the lowest level (Figure 5B). To clarify the biological functions of this lncRNA in PDAC progression, small interfering RNA (siRNA) targeting TRAF3IP2-AS1 (labeled as si-TRAF3IP2-AS1) and its non-targeting control siRNA (labeled as si-NC) were synthesized and transfected into PANC-1 and SW1990 cells. The knockdown cell models were successfully established (Figure 5C). After transfection, Cell Counting Kit-8 assays in PANC-1 and SW1990 cells revealed that knockdown of TRAF3IP2-AS1 increased the growth rates of these cell lines (Figure 5D), in line with the quantitative outcomes of the EdU experiment (Figure 5E and Supplementary Figure S6A). In addition, knockdown of TRAF3IP2-AS1 significantly strengthened the colony-formation ability of PDAC cells (Figure 5F). These results suggest that TRAF3IP2-AS1 could inhibit the growth of PDAC in vitro.

**Figure 6:**
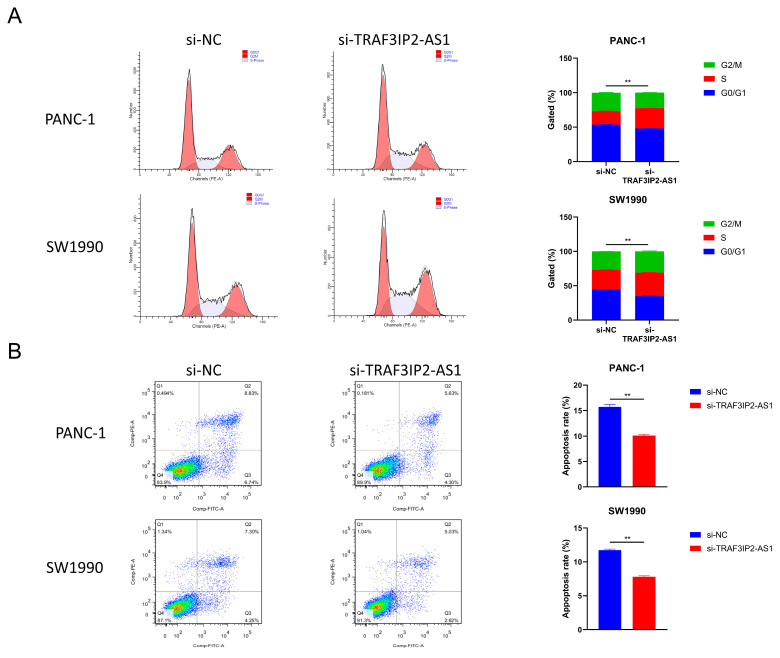
Impact of TRAF3IP2-AS1 knockdown on cell cycle and apoptosis. (**A**) Knockdown of TRAF3IP2-AS1 caused an increase in the S phase during the cell cycle. (**B**) The knockdown of TRAF3IP2-AS1 reduced the apoptosis rate of PDAC cells. The results are presented as quadrant-gated plots. Channel PE stands for propidium iodide; channel FITC stands for fluorescein isothiocyanate. The apoptosis rate exhibited in each bar chart is the sum of cell percentages in sections Q2 (late-phase apoptotic cells) and Q3 (early-phase apoptotic cells). ** *p* < 0.01.

Furthermore, changes in cell cycle were detected after TRAF3IP2-AS1 knockdown. The percentage of PDAC cells in S phase increased significantly after knockdown, meaning enhanced growth activity (Figure 6A). This finding was consistent with the alterations in proliferation ability. The apoptosis levels were also measured using flow cytometry. The experimental outcomes demonstrated that the decrease in TRAF3IP2-AS1 reduced the apoptosis of PDAC cells (Figure 6B). Additionally, the activities of caspase 3 and caspase 9 were significantly downregulated in the knockdown group (Supplementary Figure S6B,C). To sum up, lncRNA TRAF3IP2-AS1 was proved to possess an anti-tumor ability in PDAC in vitro.

### 3.7. Low Expression of TRAF3IP2-AS1 Prompted PDAC Progression In Vivo

**Figure 7:**
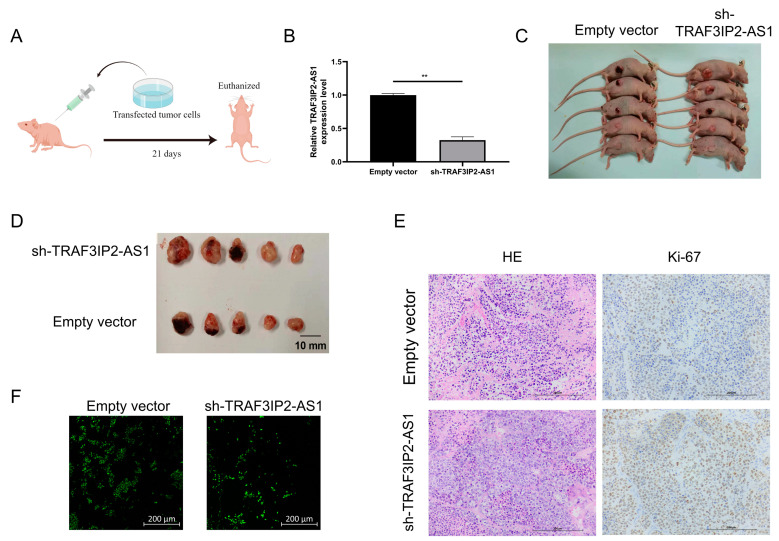
TRAF3IP2-AS1 inhibited PDAC progression in vivo. (**A**) Procedure of the in vivo experiment. (**B**) The knockdown efficacy of shRNA was proved by qRT-PCR. (**C**) Mice were euthanized 21 consecutive days after the subcutaneous injection of transfected tumor cells. (**D**) Tumors were harvested from the mice after 21 consecutive days. (**E**) Ki-67 staining showed that the proliferation was more vigorous in tumor specimens from the knockdown group. (**F**) TUNEL staining showed that the rate of apoptosis was lower within the tumor lesions from the knockdown group. ** *p* < 0.01.

In order to confirm the anti-tumor functions of TRAF3IP2-AS1 in vivo, a subcutaneous xenograft model was employed (the basic procedure is shown in Figure 7A). PANC-1 cells were transfected with short hairpin RNA (shRNA) with the assistance of lentivirus vectors. Knockdown efficacy was validated by qRT-PCR (Figure 7B) and the stable knockdown cell line was successfully established. We then injected the tumor cells subcutaneously into the Balb/c nude mice. After 21 days of growth, we found that the knockdown group presented significant growth enhancement in vivo compared with the negative control group (Figure 7C). When TRAF3IP2-AS1 had a lower expression status, the tumor sizes were larger (Figure 7D). This difference in tumor size was consistent with our findings in statistical analysis on PDAC patients from TCGA cohort (Supplementary Table S4).

Furthermore, the tumors were harvested and sliced. Ki-67 staining revealed that sh-TRAF3IP2-AS1 facilitated the proliferation of PDAC lesions (Figure 7E). The Ki-67 staining was quantitatively estimated by H score (Supplementary Figure S6D) TUNEL staining demonstrated that the level of apoptosis decreased in the knockdown group (Figure 7F, Supplementary Figure S6E). Taken together, we concluded that the low expression of TRAF3IP2-AS1 facilitated PDAC progression in vivo.

## 4. Discussion

In recent decades, increasing research has suggested that m6A modification and lncRNA play pivotal roles in areas such as immunity, inflammation and, most importantly, tumor biology [40,41,42,43,44] However, current efforts in this regard have only concentrated on single lncRNA or m6A regulator proteins [45,46]. Combinational studies based on integrated m6A and lncRNA have not been widely carried out. Determining the utility of m6A-related lncRNA expression patterns for patient subtyping, prognostic prediction and TIME feature portrayal may lead to a novel approach that could deepen our understanding of PDAC. Therefore, active effort should be invested in such research.

In our study, by utilizing sequencing data from TCGA, m6A-related lncRNA was identified using univariate Cox regression and Pearson correlation analysis. Initially, two clusters of patients were divided according to m6A-lncRNA expression pattern using unsupervised consensus clustering. Apparent differences in immune cell infiltration, functional pathway enrichment and immune-checkpoint-related molecular expression patterns were found between distinct clusters. Compared with cluster 2, cluster 1 possessed lower levels of infiltration in almost all types of immunocytes, while no significant difference was detected in stromal components. Subsequent investigation revealed that cluster 2 had a higher immune score, consistent with the infiltration analysis. Notably, cluster 2 possessed relatively higher expression levels for critical immune checkpoint molecules such as CD44 (a multifunctional player in cancer progression [47]), CD40 (a molecular target symbolizing a higher response rate to immune checkpoint blockade therapy [48]) and LGALS9 (an immunotherapy target that could regulate T cell death [49]). These findings suggest that immunotherapy is a more suitable treatment option for PDAC patients with similar m6A-lncRNA expression patterns to those in cluster 2. The preliminary analysis of m6A-lncRNA showed its value in distinguishing TIME features and immunotherapeutic responses.

Furthermore, we revealed the value of m6A-lncRNA for prognosis prediction. On the basis of the expression values of 11 prognosis-related m6A-lncRNAs, we formulated a risk score signature with the assistance of LASSO Cox regression analysis. Our risk score model showed efficacy in predicting the prognosis of PDAC patients, and we confirmed that it was an independent risk factor. The prediction model could be applied to PDAC patients under various clinicopathological conditions, demonstrating its wide adaptability. Moreover, when analyzed separately, each of the m6A-lncRNA presented prognostic prediction abilities. These findings demonstrate the important roles of m6A-lncRNA in PDAC, providing a novel and effective tool for prognosis estimation.

Furthermore, in a subsequent analysis, we found that the risk score signature correlated with TIME characteristics. The CIBERSORTx algorithm is a sharp tool for analyzing and quantifying immune infiltration based on profiling data [50]. The PDAC TIME features were estimated and scored in three dimensions: stromal score (quantification of stromal components), immune score (quantification of infiltrative immune cells) and ESTIMATE score (overall quantification of tumor component purity) [50].By interpreting the CIBERSORTx outcomes, we discovered that the risk score was only positively correlated with the abundance of M0 macrophages and activated NK cells, while it was negatively correlated with the abundance of CD8^+^ T cells, CD4^+^ T cells, plasma cells and naive B cells. The results signify that the patients with lower risk scores had a more activated TIME and more infiltrated immune cells, which may be of benefit for immunotherapy [51]. Interestingly, we found that low-risk-score patients had a higher stromal score, immune score and ESTIMATE score simultaneously. Although the infiltrative immune cells were more abundant in the low-risk-score group, a higher stromal score might have indicated that the infiltrative immune cells were more likely to be blockaded by stromal components, such as the extracellular matrix secreted by cancer fibroblasts [7,52].The penetration of these immune cells into tumor parenchyma and their anti-tumor efficacy might be weakened [52]. We also revealed the pervasive negative correlation between the risk score signature and immune checkpoint blockade genes in PDAC patients. Patients with lower risk scores were more suitable for immune checkpoint blockade therapies. These results prove that our risk score signature was effective in portraying TIME and guiding immunotherapy in PDAC patients.

TRAF3IP2-AS1 is a hub m6A-lncRNA with a dysregulated expression pattern in the panel. As the anti-sense sibling of *TRAF3IP2*, TRAF3IP2-AS1 was initially recognized in non-tumor pathological conditions, such as cocaine abuse, mental disorders and auto-immune disease [53,54,55]. Investigation of its role in cancer is limited. One bioinformatic analysis showed that it could be used as a biomarker in glioblastoma progression, while another study revealed that TRAF3IP2-AS1 propelled the development of a specific type of renal cancer (NONO-TFE3 translocation type) via PTEN downregulation and PARP1 m6A stimulation [56,57]. However, its clinical utility in PDAC remains to be seen. We revealed the downexpression of TRAF3IP2-AS1 in PDAC lesions, which was related to worse prognosis and larger tumor size. Further investigations demonstrated that TRAF3IP2-AS1 could hinder the proliferation of PDAC tumors in vitro and in vivo. The knockdown of TRAF3IP2-AS1 led to decreased apoptosis and alterations of cell cycle distribution, proving that this lncRNA possessed anti-tumor properties. Notably, we observed that after knockdown, the activities of caspase 3 and caspase 9 were significantly reduced, indicating TRAF3IP2-AS1 might affect cellular apoptosis via the mitochondrial pathway in PDAC. The conclusions of in vitro and in vivo experiments were consistent with the bioinformatic findings and clinical statistical outcomes (Supplementary Table S4).

In recent years, m6A has become a hot topic in tumor biology [58,59]. Generally speaking, current research on m6A in PDAC is still at an early stage [45,46]. Nevertheless, emerging evidence has revealed that m6A regulator proteins are involved in the carcinogenesis, progression and therapy resistance of PDAC [32,33,34,35,36,46]. However, the biological roles of m6A-related lncRNAs in PDAC are rarely explored. Therefore, the findings of our research fill in some of the blanks in this area. We demonstrated the research value and potential of m6A-lncRNA in PDAC, which may help in the integration of the m6A regulation network in PDAC.

However, there were some limitations to our study. First, all analyses of m6A-lncRNAs were performed based on TCGA and bioinformatic algorithms because of technical limitations. Second, no large-scale or multi-center clinical cohorts or samples were used to validate the clinical utility and efficacy of m6A-lncRNAs. Third, we only elucidated the functional characterization of m6A-lncRNA TRAF3IP2-AS1 in vivo and in vitro; the specific mechanisms and internal interactions remain unknown. Therefore, further research in a large-scale clinical cohort and more detailed mechanistic studies are needed.

## 5. Conclusions

In conclusion, we analyzed the expression pattern of m6A-lncRNAs in 177 PDAC patients. The distinct subgroups clustered by expression pattern corresponded to different TIME characteristics, outcomes and enriched biological pathways. We also established a prognostic model based on 11 m6A-lncRNAs. The model was demonstrated to be efficient and accurate in predicting PDAC outcomes and could be applied to patients with different clinicopathological characteristics. The m6A-lncRNA model could also depict the different TIME features and be used to guide immunotherapies. After bioinformatics analysis, we selected TRAF3IP2-AS1 (a hub m6A-lncRNA in our model) and clarified its anti-tumoral functions in PDAC development. Our findings will help clinicians improve the outcomes of PDAC. Further studies with larger clinical cohorts and samples should be carried out to confirm the efficacy of our model and to explore the specific mechanisms of m6A-lncRNAs at a deeper level.

## Figures and Tables

**Table 1:** M6A regulators included in analysis.

Classification	Regulator Name
Writers	VIRMA, LRPPRC, WTAP, ZC3H13, METTL3, RBM15/15B/X, METTL14, METTL16
Readers	IGFBP2, FMR1, HNRNPA2B1, YTHDF1, YTHDC2, YTHDF3, HNRNPC, IGFBP1, IGFBP3, YTHDF2, YTHDC1
Erasers	ALKBH5, FTO

## Data Availability

The public datasets in this study can be obtained from TCGA database https://portal.gdc.cancer.gov/ (accessed on 10 January 2022) and the GTEx database https://www.gtexportal.org/ (accessed on 23 January 2022).

## Supplementary Materials

The following supporting information can be downloaded at: https://www.mdpi.com/article/10.3390/vaccines11030499/s1, Figure S1: M6A regulator genes were dysregulated in PDAC. (A) The expression levels of all 23 m6A regulators were significantly different between normal samples and tumor samples from TCGA and GTEx cohorts. (B) qRT-PCR validation of difference in expression using clinical tissue specimens. Figure S2. The results of the NMF algorithm. (A) The NMF algorithm indicators corresponding to k = 2 to k = 9. (B) The results of consensus clustering corresponding to k = 2 to k = 9. Identification of 11 hub prognostic m6A-lncRNAs. Figure S3. Identification of 11 hub prognostic m6A-lncRNAs. (A) Spectrum of the LASSO coefficient on 66 prognosis-related m6A-lncRNA. (B) Identification of the best penalty parameters λ in the LASSO algorithm using ten-fold crossing validation. The dotted line on the left indicates the best λ value and the minimum error point, which corresponds to the best number of lncRNAs selected in the model. (C) Risk curve presenting the distribution of risk score distribution and corresponding survival situations of PDAC cases from the training set. (D) Risk curve presenting the risk score distribution and survival situations of PDAC cases from the testing set. Figure S4. Expression level of each lncRNA correlated with the survival rate of PDAC patients. (A) K-M analysis revealing high AC005332.4 expression correlated with better prognosis. (B) K-M analysis revealing high AC078923.1 expression correlated with better prognosis. (C) K-M analysis revealing high AC090114.2 expression correlated with better prognosis. (D) K-M analysis revealing high AC245140.1 expression correlated with better prognosis. (E) K-M analysis revealing high LINC01091 expression correlated with better prognosis. (F) K-M analysis revealing high LINC01954 expression correlated with better prognosis. (G) K-M analysis revealing high LINC02044 expression correlated with better prognosis. (H) K-M analysis revealing high MEG9 expression correlated with better prognosis. (I) K-M analysis revealing high MIR3142HG expression correlated with better prognosis. (J) K-M analysis revealing high TRAF3IP2-AS1 expression correlated with better prognosis. (K) K-M analysis revealing high TRPC7-AS1 expression correlated with better prognosis. Figure S5. Stratification analysis of risk score signature according to clinicopathological indexes. (A) K-M analysis demonstrating prognosis was significantly different according to risk score in patients with age <= 65. (B) K-M analysis demonstrating prognosis was significantly different according to risk score in patients with age > 65. (C) K-M analysis demonstrating prognosis was significantly different according to risk score in female patients. (D) K-M analysis demonstrating prognosis was significantly different according to risk score in male patients. (E) K-M analysis demonstrating prognosis was significantly different according to risk score in patients with tumor stage G1-2. (F) K-M analysis demonstrating prognosis was significantly different according to risk score in patients with tumor stage G3-4. (G) K-M analysis demonstrating prognosis was significantly different according to risk score in patients with tumor stage T1-2. (H) K-M analysis demonstrating prognosis was significantly different according to risk score in patients with tumor stage T3-4. (I) K-M analysis demonstrating prognosis was significantly different according to risk score in patients with tumor stage N0. (J) K-M analysis demonstrating prognosis was significantly different according to risk score in patients with tumor stage N1. (K) K-M analysis demonstrating prognosis was significantly different according to risk score in patients with tumor stage M0. (L) K-M analysis demonstrating prognosis was significantly different according to risk score in patients with tumor stage M1. Figure S6. Quantification of EdU, caspase 3/9 activity, Ki-67 and TUNEL assays. (A) The percentage of EdU positive cells in the total cells. (B,C) Changes in caspase3 and caspase 9 activities after TRAF3IP2-AS1 knockdown were determined. (D) The H Score of Ki-67 slides. (E) The TUNEL positive percentages were calculated and compared.; Table S1: The primer sequences used for qRT-PCR. Table S2. The sequences of siRNA for TRAF3IP2-AS1. Table S3. The target sequences of shRNA for TRAF3IP2-AS1. Table S4. Statistical correlation between expression levels of TRAF3IP2-AS1 and clinicopathological characteristics of PDAC patients.
