# Supplementary material for: M6A-Related Long Non-Coding RNA Displays Utility in Predicting Prognosis, Portraying the Tumor Immune Microenvironment and Guiding Immunotherapy in Pancreatic Ductal Adenocarcinoma
Source: Vaccines (Basel). 2023 Feb 21;11(3):499. doi: 10.3390/vaccines11030499 (PMC10056289; doi:10.3390/vaccines11030499)
Supplement: Supplementary file 1 [file vaccines-11-00499-s001.zip › vaccines-2194642-supplementary/Supplementary Figures.pdf]

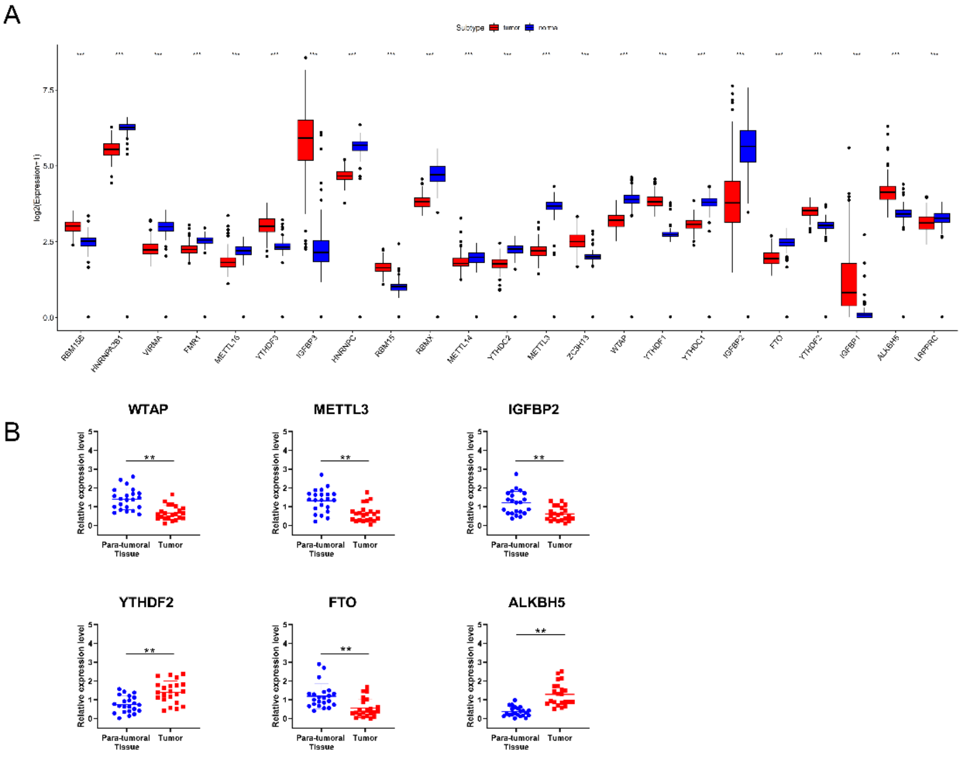

Figure S1. M6A regulator genes were dysregulated in PDAC(A) The expression levels of all 23 m6A regulators were significantly different between normal samples and tumor samples from TCGA and GTEx cohorts. (B) qRT-PCR validation of difference in expression using clinical tissue specimens.

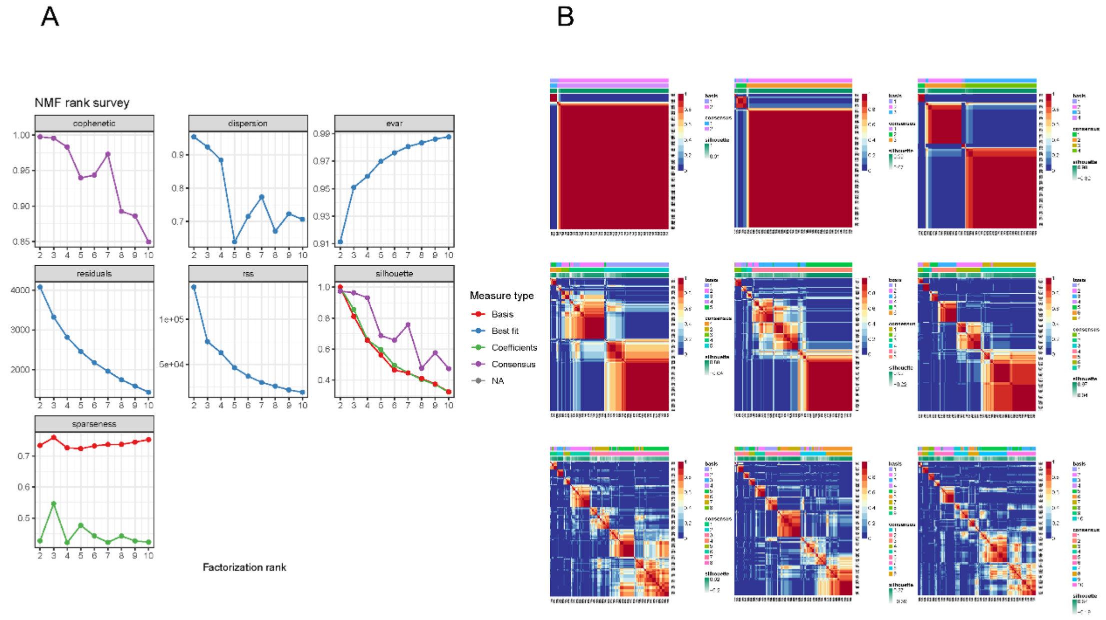

Figure S2. The results of the NMF algorithm. (A) The NMF algorithm indicators corresponding

to k=2 to k=9. **(B)** The results of consensus clustering corresponding to k=2 to k=9.

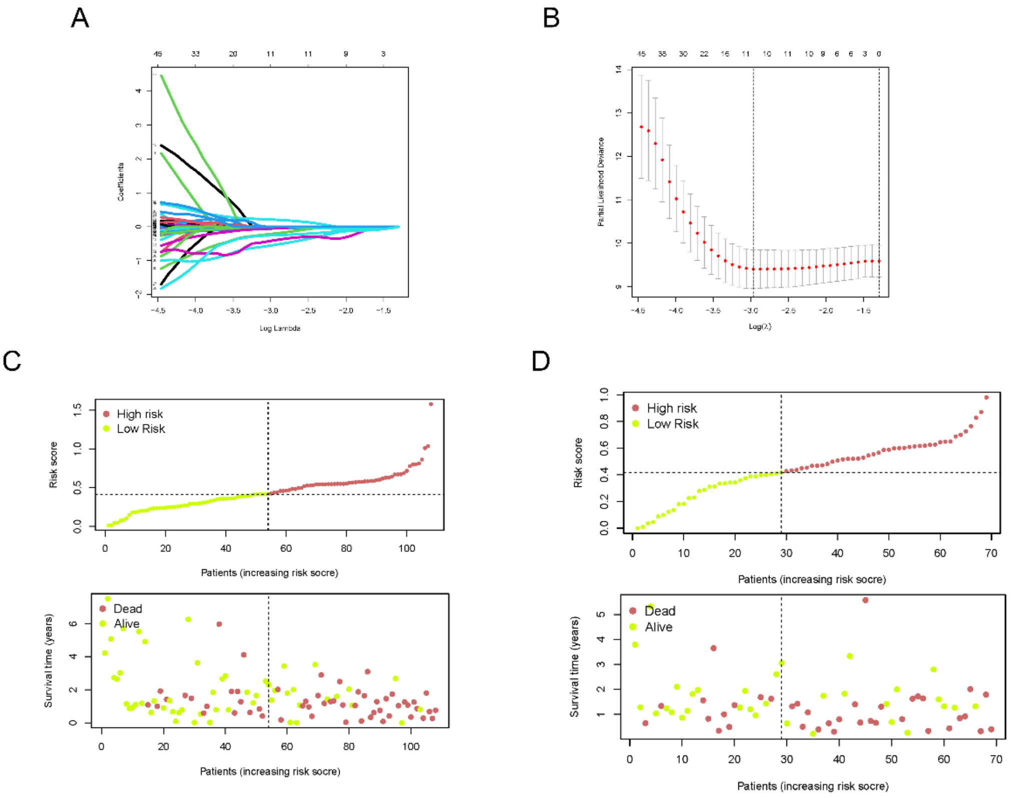

Figure S3. Identification of 11 hub prognostic m6A-lncRNAs. (A) Spectrum of the LASSO coefficient on 66 prognosis-related m6A-lncRNA. (B) Identification of the best penalty parameters λ in the LASSO algorithm using ten-fold crossing validation. The dotted line on the left indicates the best λ value and the minimum error point, which corresponds to the best number of lncRNAs selected in the model. (C) Risk curve presenting the distribution of risk score distribution and corresponding survival situations of PDAC cases from the training set. (D) Risk curve presenting the risk score distribution and survival situations of PDAC cases from the testing set.

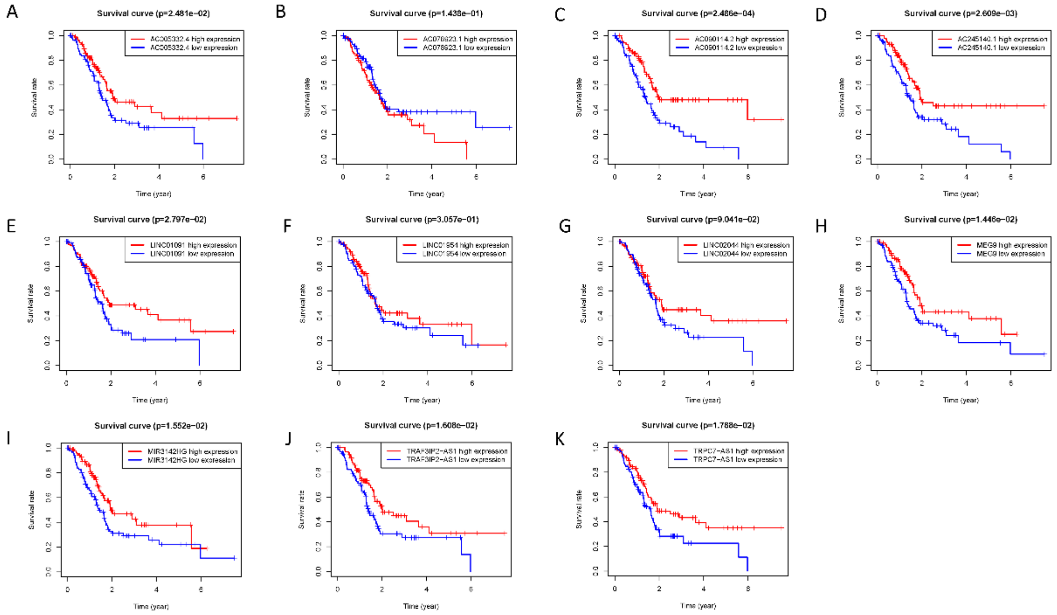

Figure S4. Expression level of each lncRNA correlated with the survival rate of PDAC patients(A) K-M analysis revealing high AC005332.4 expression correlated with better prognosis. (B) K-M analysis revealing high AC078923.1 expression correlated with better prognosis. (C) K-M analysis revealing high AC090114.2 expression correlated with better prognosis. (D) K-M analysis revealing high AC245140.1 expression correlated with better prognosis. (E) K-M analysis revealing high LINC01091 expression correlated with better prognosis. (F) K-M analysis revealing high LINC01954 expression correlated with better prognosis. (G) K-M analysis revealing high LINC02044 expression correlated with better prognosis. (H) K-M analysis revealing high MEG9 expression correlated with better prognosis. (I) K-M analysis revealing high MIR3142HG expression correlated with better prognosis. (J) K-M analysis revealing high TRAF3IP2-AS1 expression correlated with better prognosis. (K) K-M analysis revealing high TRPC7-AS1 expression correlated with better prognosis.

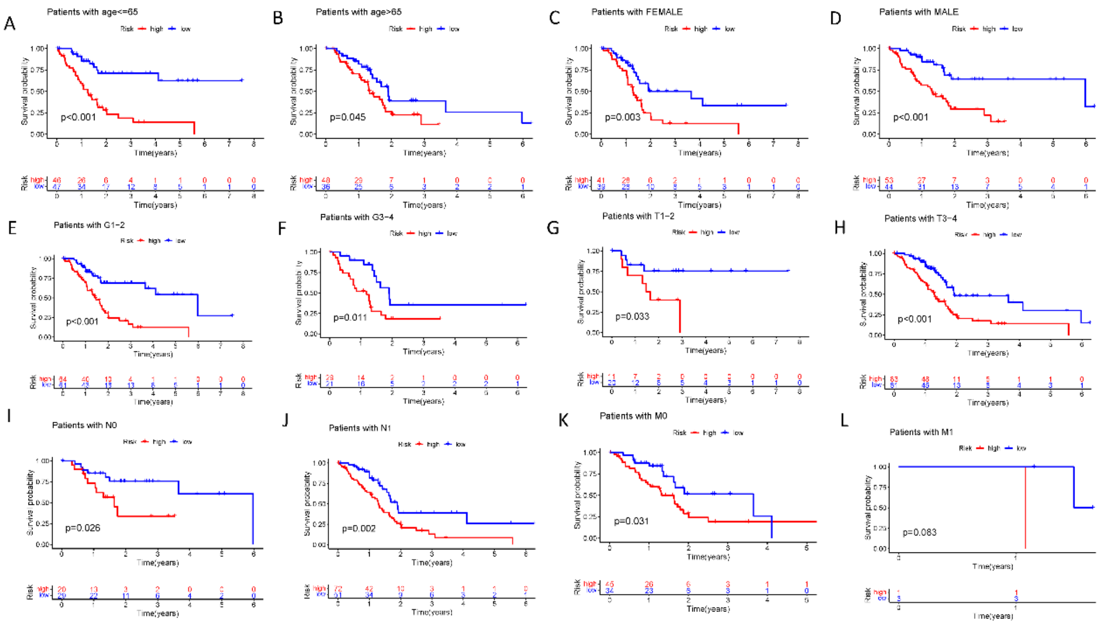

Figure S5. Stratification analysis of risk score signature according to clinicopathological indexes. (A) K-M analysis demonstrating prognosis was significantly different according to risk score in patients with age <= 65. **(B)** K-M analysis demonstrating prognosis was significantly different according to risk score in patients with age > 65. **(C)** K-M analysis demonstrating prognosis was significantly different according to risk score in female patients. **(D)** K-M analysis demonstrating prognosis was significantly different according to risk score in male patients. **(E)** K-M analysis demonstrating prognosis was significantly different according to risk score in patients with tumor stage G1-2. **(F)** K-M analysis demonstrating prognosis was significantly different according to risk score in patients with tumor stage G3-4. **(G)** K-M analysis demonstrating prognosis was significantly different according to risk score in patients with tumor stage T1-2. **(H)** K-M analysis demonstrating prognosis was significantly different according to risk score in patients with tumor stage T3-4. **(I)** K-M analysis demonstrating prognosis was significantly different according to risk score in patients with tumor stage N0. **(J)** K-M analysis demonstrating prognosis was significantly different according to risk score in patients with tumor stage N1. **(K)** K-M analysis demonstrating prognosis was significantly different according to risk score in patients with tumor stage M0. **(L)** K-M analysis demonstrating prognosis was significantly different according to risk score in patients with tumor stage M1.

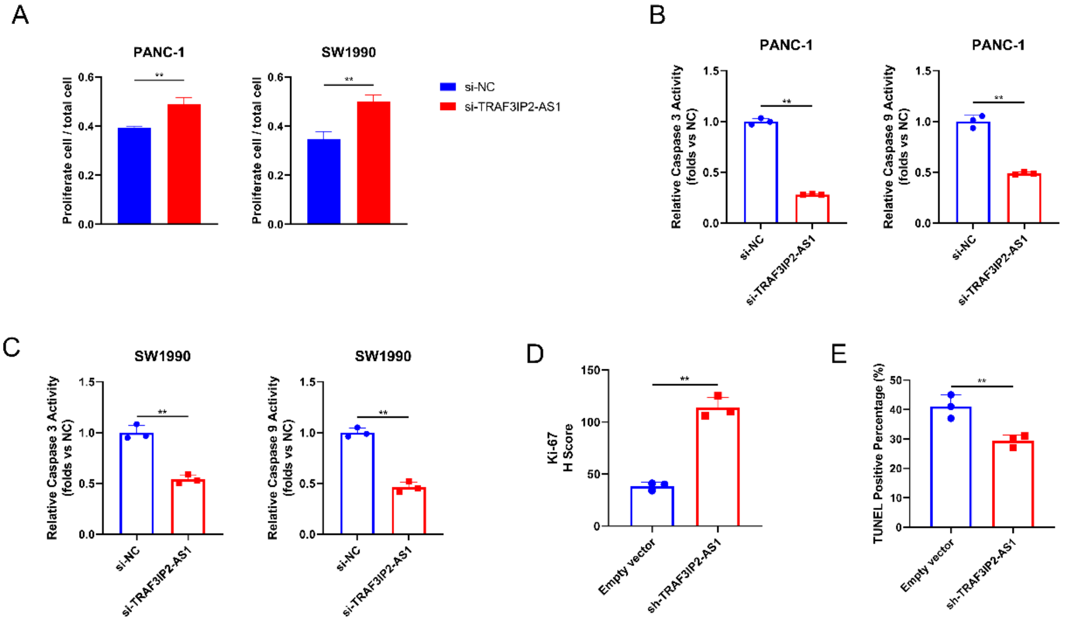

Figure S6. Quantification of EdU, caspase 3/9 activity, Ki-67 and TUNEL assays. (A) The percentage of EdU positive cells in the total cells. **(B-C)** Changes in caspase3 and caspase 9 activities after TRAF3IP2-AS1 knockdown were determined. **(D)** The H Score of Ki-67 slides. **(E)** The TUNEL positive percentages were calculated and compared.
