# Supplementary material for: M6A-Related Long Non-Coding RNA Displays Utility in Predicting Prognosis, Portraying the Tumor Immune Microenvironment and Guiding Immunotherapy in Pancreatic Ductal Adenocarcinoma
Source: Vaccines (Basel). 2023 Feb 21;11(3):499. doi: 10.3390/vaccines11030499 (PMC10056289; doi:10.3390/vaccines11030499)
Supplement: Supplementary file 1 [file vaccines-11-00499-s001.zip › vaccines-2194642-supplementary/Supplementary Tables.pdf]

Table S1. The primer sequences used for qRT-PCR

Gene	Forward primer (5'→3')	Reverse primer (5'→3')
TRAF3IP2-AS1	TTTGGCGGCTATGCAGGATT	TGTCCATGTGGTATTGGGCA
WTAP	ACTGGCCTAAGAGAGTCTGAAG	GTTGCTAGTCGCATTACAAGGA
METTL3	TTGTCTCCAACCTTCCGTAGT	CCAGATCAGAGAGGTGGTGTAG
IGFBP2	GACAATGGCGATGACCACTCA	CAGCTCCTTCATACCCGACTT
YTHDF2	CCTTAGGTGGAGCCATGATTG	TCTGTGCTACCCAACTTCAGT
ALKBH5	CGGCGAAGGCTACACTTACG	CCACCAGCTTTTGGATCACCA
FTO	ACTTGGCTCCCTTATCTGACC	TGTGCAGTGTGAGAAAGGCTT
GAPDH	AACGGATTTGGTCGTATTGGG	CCTGGAAGATGGTGATGGGAT

Table S2. The sequences of siRNA for TRAF3IP2-AS1 knockdown

Name	Forward sequence (5'→3')	Reverse sequence (5'→3')
siRNA	GGAGUAUGUGCUUAAGGAAAU	UUCCUUAAGCACAUACUCCAA

Table S3. The target sequences of shRNA for TRAF3IP2-AS1 knockdown

Name	Target sequence (5'→3')
shRNA 1	GCAACGCCGCAGTGTTTATAA
shRNA 2	GGCACACATGAACAGACTTCA
shRNA 3	GCACTGACTGGTTCAGCTATT

Table S4. Statistical correlation between expression levels of TRAF3IP2-AS1 and clinicopathological characteristics of PDAC patients.

Clinical characteristics	TRAF3IP2-AS1 Expression		*P* value
	Low (n=87)	High (n=87)	
**Age(years)**			
<65	40	39	0.879
≥65	47	48	
**Gender**			0.36
Male	51	45	

Female	36	42	
**Grade**			
G1-2	56	66	0.093
G2-4	30	20	
Unknown	1	1	
**T**			
T 1~2	8	22	0.005
T 3~4	79	65	
**N**			
N0	23	25	0.770
N1	62	61	
Nx	2	1	
**Metastasis**			
Yes	41	39	0.961
No	2	2	
Unknown	44	46	
